# Trajectories and associations between wearable-derived mobility, patient-reported outcomes, clinical measures and clinical indicators following total knee arthroplasty: The IMPACT project protocol

**DOI:** 10.1371/journal.pone.0345667

**Published:** 2026-04-15

**Authors:** Daniel Hickey, Rory Lambe, Quentin Le Cornu, Anthony Pierce, Ian Byrne, Cailbhe Doherty, Brian Caulfield

**Affiliations:** 1 School of Public Health, Physiotherapy and Sports Science, University College Dublin, Dublin, Ireland; 2 Insight Research Ireland Centre for Data Analytics, University College Dublin, Dublin, Ireland; 3 Beacon Orthopaedic Centre, Beacon Hospital, Sandyford, Dublin, Ireland; 4 CSTAR Centre for Support and Training in Analysis and Research, University College Dublin, Dublin, Ireland; Northwestern University Feinberg School of Medicine, UNITED STATES OF AMERICA

## Abstract

**Background:**

Total Knee Arthroplasty (TKA) is one of the most frequently performed elective surgical procedures worldwide. In the United States, annual procedure volumes are projected to exceed 3.5 million by 2030, while demand in Ireland is expected to rise by 49% by 2036. Despite its overall effectiveness, up to 20% of patients remain dissatisfied post-operatively. Conventional follow-up methods rely on intermittent Patient-Reported Outcome Measures (PROMs) and clinic-based performance tests, which provide only a partial view of recovery. Wearable-derived mobility data offers the opportunity to capture daily activity patterns in free-living conditions, complementing PROMs and clinical assessments to refine understanding of recovery trajectories following TKA.

**Methods:**

This prospective observational cohort study will recruit up to 160 participants scheduled for unilateral TKA at the Beacon Hospital (Dublin, Ireland). Participants will wear a Garmin Vivosmart 5 continuously from up to four weeks pre-operatively to 6 months post-operatively. Continuous, minute-level wearable data will be collected including daily step count. PROMs will be collected using the Labfront Companion mobile application and will include weekly numeric ratings of pain, fatigue, stiffness and sleep quality; the Oxford Knee Score (OKS) at 30-day intervals; and the EQ-5D-5L at 60-day intervals. Clinical indicators and measures will be abstracted from the electronic health record (Meditech). Clinical and demographic measures include age, sex, height, weight, body mass index (BMI), and participation in prehabilitation exercise classes. Primary statistical analysis will evaluate longitudinal trend analysis of step count and PROMs over time after surgery, while covariate-adjusted Spearman-type correlations will evaluate cross-sectional associations between daily step count, OKS, EQ-5D-5L and pain.

**Expected outcomes:**

This study will characterise wearable-derived mobility metrics across early and mid-stage recovery following TKA, and evaluate their associations with PROMs.

**Conclusions:**

Integrating continuous wearable data with PROMs and clinical information may refine post-operative monitoring and support personalised rehabilitation following TKA.

## Introduction

Osteoarthritis (OA) is a degenerative joint disease characterised by pain, stiffness, swelling, and progressive loss of mobility [1]. It affects an estimated 365 million people worldwide, most commonly at the knee [1]. With an ageing population and increasing rates of obesity and injury, the burden of knee osteoarthritis is projected to rise further [1]. Total Knee Arthroplasty (TKA) is generally regarded as the gold-standard surgical intervention for end-stage knee OA [2]. As the burden of knee OA rises, the demand for TKA also increases. In the United States, annual TKA procedure volumes are projected to increase by 36% by 2060 [3], while in Ireland demand is projected to rise by 49% by 2036 [4].

Despite its frequency, up to 20% of patients remain dissatisfied following TKA [5–7], most often due to persistent pain, limited functional improvement, or unmet expectations [7]. Increased physical activity is a common goal for patients following TKA, yet research has shown minimal or inconsistent improvements in objectively measured physical activity six to twelve months postoperatively [8]. A more thorough understanding of how activity levels evolve throughout recovery following TKA will enable tailored rehabilitation strategies and help manage patient expectations.

Post-operative recovery following TKA is commonly evaluated using Patient-Reported Outcome Measures (PROMs). Validated PROMs include the Oxford Knee Score (OKS) and EuroQol Five Dimension EQ-5D-5L [9]. While PROMs are often central to patient-centred care following TKA, they are subject to recall bias, response bias and are limited by ceiling and floor effects [10,11]. Emerging evidence suggests that improvements in PROMs may not reliably reflect objective functional recovery. In a prospective cohort study of 152 TKA patients, Wu et al. reported weak correlations (r = 0.2–0.3) between changes in PROMs and in gait-related metrics such as step count following surgery [12].

Functional performance tests, such as the Timed Up and Go (TUG) and 6-Minute Walk Test (6MWT), provide objective assessments of mobility and balance but are performed infrequently and under controlled clinical conditions, limiting their ability to capture variability and progression of recovery in patient’s daily environments [11].

The growing availability of wrist-worn wearable devices has enabled continuous, passive collection of physical activity metrics in free-living conditions [13,14]. These devices can capture step count, cadence, activity duration, and other real-time markers of mobility, providing a more accurate representation of daily recovery [11]. In the context of this study, patient mobility refers specifically to real-world ambulatory behaviour, operationalised primarily through wearable-derived step count.

Despite advances in wearable technology, most existing studies in this field are limited to short-term follow-up or laboratory-based protocols, and few have integrated wearable-derived mobility data with PROMs and clinical indicators to build a multidimensional model of recovery [15–17].

The present study addresses these gaps by using wrist-worn accelerometers to continuously track patient mobility from up to four weeks pre-operatively through to six months post-operatively in individuals undergoing elective TKA. PROMs, clinical indicators and clinical and demographic measures will be integrated to contextualise mobility trends. This longitudinal dataset will enable nuanced insights into mobility recovery patterns following TKA and their associations with PROMs. This study also aims to assess the temporal associations between early outcome measures and medium-term outcome measures following TKA. This manuscript describes the study protocol for The IMPACT Project (Integrating Mobility, Patient-Reported Outcomes and Clinical Measures following Total Knee Arthroplasty) an internal framework designed to examine real-world postoperative recovery following TKA using wearable-derived mobility data, PROMs, and clinical information.

**Objectives:** To characterise peri-operative mobility trajectories using wearable-derived metrics, evaluate their associations with PROMs, clinical indicators and clinical and demographic measures following TKA.

## Materials and methods

### Status and timeline

The study protocol was preregistered on OSF Registries on 25 November 2025 (https://doi.org/10.17605/OSF.IO/BACX4). The protocol corresponds to Version 1.0, issued on 27 November 2025. Ethical approval for this study was granted by the Beacon Hospital Research Ethics Committee (BHREC) on 9 July 2025 (Reference BEA0246). The study is currently in the early stages of its recruitment phase and no results have been generated. Recruitment for this study commenced on 25 July 2025. The first participant was onboarded and data collection for this study commenced on 1 August 2025. Both recruitment and data collection will cease on the 1 August 2027 or when a satisfactory number of participants have been enrolled, whichever occurs first. All participants are provided with a patient information leaflet and informed consent form prior to the study. To participate, participants must provide written informed consent using the study consent form. Results for this study are expected by January 2028.

### Study design

This is a single-centre, prospective observational cohort study conducted within the IMPACT Project, an internal research framework embedded within routine elective TKA care at the Beacon Hospital, Dublin, Ireland. The study was submitted for ethical approval to the Beacon Hospital Research Ethics Committee (BHREC) on 14 Feb 2025. Following review and addressing queries, the study was approved on 9 July 2025. The study was registered on the Open Science Framework on 21 October 2025.

#### Eligibility criteria and recruitment.

The eligibility criteria (Table 1) restrict the cohort to patients undergoing unilateral TKA as a treatment for OA, thereby reducing clinical heterogeneity that could confound recovery trajectories. Exclusion of non-OA indications, revision procedures, and individuals unable to reliably engage with the wearable ensures a sample with sufficient capacity for sustained device adherence and valid longitudinal mobility measurement. To ensure adequate baseline data collection, patients are required to be enrolled at least 7 days prior to surgery. Patients outside a four-week window relative to their surgery date will not be included.

**Table 1:** Eligibility criteria.

Inclusion Criteria	Exclusion Criteria
Patients referred to the Beacon Hospital for TKA as treatment for OA Adults scheduled for TKA who are ≥ 7 days pre-operative Participants aged 18 years and older	Individuals lacking the capacity to consent Persons <18 years of age Those undergoing TKA due to cancer, trauma, avascular necrosis, or bone growth disorders Patients requiring revision TKA Individuals with significant neurological or psychiatric conditions that could interfere with the effective use of the wearable device

#### Study size.

A study sample of up to 160 participants will be sought over the two-year recruitment period. Given the exploratory nature of this study, no formal sample size or power calculation was performed. The planned study size was determined pragmatically based on feasibility, anticipated recruitment rates and previous orthopaedic studies using wearable devices [17].

#### Data collection.

Wearable-derived mobility data will be captured using the Garmin Vivosmart 5, which streams minute-level step count data through the Labfront platform. Step counts are generated from Garmin’s proprietary accelerometer-based step-detection algorithms. From these data, higher-order metrics will be derived to characterise post-operative mobility recovery, including daily step count, step-positive minutes (number of minutes with ≥1 recorded step), activity window length (time between first and last step-positive minute), longest inactivity streak (maximum consecutive zero-step minutes), diurnal activity distribution (steps per hour and AM:PM ratio), and day-to-day variability (standard deviation and coefficient of variation of daily steps within rolling 7-day windows).

Sleep duration, resting heart rate (RHR) and heart rate variability (HRV) will be retained as exploratory secondary measures rather than core outcomes. Night-time wear is optional to reduce participant burden. For participants who choose to wear the device overnight, sleep duration will be derived from actigraphy-based algorithms integrating movement and optical heart-rate signals. RHR and HRV will be passively collected through the Vivosmart 5’s wrist-worn photoplethysmography sensor and processed through Garmin’s proprietary algorithms using the Labfront platform. In a laboratory validation study, Garmin devices demonstrated over-estimation of sleep duration by an average of 44 minutes, fair agreement for heart rate (ICC 0.41) with wide error margins (–20 to +30 bpm), poor HRV performance with substantial bias (–22.4 Ms), wide limits of agreement (±92 Ms) and low reliability (ICC 0.24) compared with ECG-derived HRV [18]. These findings indicate that sleep, RHR and HRV lack the stability and validity required for core study outcomes.

However, RHR and HRV provide numeric representations of general physiological function [19], with HRV is recognised as an indicator of autonomic nervous system activity [20]. Therefore, fluctuations in these signals may help contextualise unusual mobility patterns, such as transient reductions in step count during periods of illness or physiological stress. Additionally, poor sleep is associated with mental health disorders and pain sensitization [21,22], both of which may affect post-operative mobility.

PROMs will be collected electronically using the Labfront mobile platform. Onboarding will include installation of the Labfront Companion application and patient education on PROM completion. Schedule and procedure for PROM collection is summarised in Table 2.

From every Saturday post-onboarding to Day 210, participants will complete weekly questionnaires on pain, stiffness, fatigue, and sleep quality using a 0–10 Numerical Rating Scale (NRS). These PROMs will refer to the participant’s experience over the previous 7 days (e.g., “How fatigued have you felt over the last 7 days on a scale of 0 to 10, where 0 indicates no fatigue and 10 indicates maximal fatigue”). Participants will receive a push notification at 9am to complete the four questionnaires, if the questionnaires have not been completed by 7 pm that evening, participants will receive a reminder push notification. Questionnaires close at 9 pm every Saturday evening and will vanish from participant dashboards on the Labfront Companion application.

The OKS will be administered at 30-day intervals, beginning on Day 1 post-onboarding and repeated on Days 30, 60, 90, 120, 150, 180, and 210. The EQ-5D-5L will be administered at 60-day intervals on Days 1, 60, 120, and 180. For both instruments, participants will receive automated push notifications at 9am on the scheduled assessment day, and questionnaires will remain accessible for a 7-day completion window.

This schedule is designed to capture short-term fluctuations in symptoms, medium-term changes in joint-specific function, and longer-term improvements in health-related quality of life, and is consistent with national joint registry practice [23]. All PROMs will be time-stamped and monitored for completion within the Labfront platform, which issues automated reminders and records compliance.

Clinical indicators will be abstracted from the electronic medical record (Meditech). These will include: (i) comorbidities as listed in the preoperative assessment; (ii) surgical method (manual vs robotic assisted) and prosthesis characteristics; (iii) hospital length of stay (LOS) (iv) post-operative complications (superficial or deep infection, wound dehiscence, delayed healing); (v) adverse events (venous thromboembolism [DVT/PE], cardiovascular events [myocardial infarction, arrhythmia], pneumonia, urinary tract infection, in-hospital fall, post-operative delirium); and (vi) reinterventions (joint aspiration or injection, manipulation under anaesthesia, washout or debridement, any reoperation, revision referral, or revision surgery). These variables will be coded as binary or categorical indicators with event dates where applicable. They will be used in both descriptive and adjusted analyses.

Clinical and demographic measures will be collected during onboarding and cross-checked against the electronic medical record. These will include age, sex, height (cm, assessed using a stadiometer), weight (kg, assessed using calibrated scales), body mass index (BMI, calculated as kg/m²) and participation in prehabilitation exercise classes. An overview of data categories and measures collected is presented in Table 3.

Baseline covariates used in this statistical analysis will include age, sex, BMI, baseline OKS, baseline EQ-5D-5L, baseline pain, hospital LOS, surgical method (manual vs robotic assisted) and participation in prehabilitation exercise classes.

**Table 2:** Schedule and procedures for PROM collection.

Patient-Reported Outcome Measures	Domains Assessed	Schedule
NRS	Pain, stiffness, fatigue, sleep quality	Weekly intervals on Days 1 through to Day 210
OKS	Joint-specific pain and function	30-day intervals on days 1, 30, 60, 90, 120, 150, 180, 210
EQ-5D-5L	Health-related quality of life questionnaire	60-day intervals on days 1, 60, 120, 180

**Table 3:** Overview of data categories and measures collected in the study.

Data category	Measures collected	Source/method	Frequency
Wearable-derived mobility metrics	Daily step count (primary outcome); step-positive minutes; activity window length; longest inactivity streak; diurnal activity distribution (steps per hour, AM:PM ratio); day-to-day variability (SD and CV of daily steps); weekly rolling mean and slope; recovery dip frequency	Garmin Vivosmart 5 through the Labfront Platform	Continuous
Wearable-derived rest-activity metrics	Activity counts per minute; movement frequency (zero-crossing count); minutes above movement threshold; rest-activity ratio; diurnal activity profile; relative amplitude (RA); interdaily stability (IS); intradaily variability (IV)	Garmin Vivosmart 5 through the Labfront Platform	Continuous (minute-level data aggregated to hourly and daily summaries)
PROMs	Pain, fatigue, stiffness, sleep quality (NRS), OKS and EQ-5D-5L	Labfront Platform	Baseline/Weekly/30-day intervals/60-day intervals
Clinical indicators	Comorbidities, surgical method, prosthesis characteristics; hospital LOS; post-operative complications, adverse events, reinterventions.	Meditech electronic medical record	Accessed following termination of data collection
Clinical and Demographic Measures	Age, sex, height, weight, BMI, participation in prehabilitation	Onboarding assessment and Meditech electronic medical record cross-check	Collected at Baseline

## Statistical analysis

The primary analytical focus is descriptive characterisation of recovery patterns over time and cross-sectional associations between wearable-derived mobility metrics and PROMs. All analyses described below are exploratory in nature. The distinction between primary and secondary analyses reflects analytical focus rather than inferential priority. An overview of the statistical analysis plan can be seen in Table 4.

### Primary analyses

Descriptive analysis will be performed within the total study population. Continuous variables will be summarised as mean ± standard deviation (SD) or median (interquartile range), depending on distribution, and categorical variables as count (percentage).

All wearable-derived data will be aggregated into surgery-anchored time bins defined relative to each participant’s date of surgery (e.g., post-operative Week 1 = Days 1–7; Week 2 = Days 8–14). Prespecified 7-day analytic windows will be used for cross-sectional analyses at the following post-operative intervals: Days 1–7, 60–67, 120–127 and 180–187. These windows correspond directly to periods during which the OKS, pain NRS and EQ-5D-5L questionnaires are all completed. Aligning mobility measurement to these PROM windows will reduce temporal misalignment and increase the interpretability of associations.

Analysis of the Main Criteria:

Recovery after surgery will be assessed using four outcomes:

1. Daily step count
2. OKS
3. EQ-5D-5L
4. Pain (NRS)

Daily step counts will be obtained using a wrist-worn Garmin wearable, which has shown acceptable accuracy for ambulatory monitoring in free-living conditions [24].

To accommodate non-linear patterns in recovery over time, restricted cubic splines will be incorporated as fixed effects within mixed-effects regression models. These splines model population-level time effects and do not define individual-level or latent recovery trajectories. Participant-level random intercepts will be included to account for within-subject correlation.

For OKS and EQ-5D-5L questionnaires, random-effects Tobit models will be used to accommodate ceiling and floor effects commonly observed in health status instruments [25,26]. EQ-5D-5L utilities transformed to a 0–1 scale will additionally be analysed using beta regression [25,26].

For pain NRS, linear mixed-effects models will be used initially; if distributional assumptions are violated, mixed-effects ordinal logistic regression will be applied.

Daily step count data will be log-transformed if distributions are sufficiently right-skewed to violate model assumptions. If assumptions remain violated after transformation, alternative generalised linear mixed-effects models will be considered, guided by residual diagnostics, model fit and consistent with prior step-count modelling recommendations [24].

Predicted mean trajectories with 95% confidence intervals will be plotted for interpretation.

Cross-sectional associations between wearable-derived mobility metrics and PROM scores will be analysed within the predefined 7-day post-operative windows. Mobility data will be aggregated over the prespecified 7-day period.

Covariate-adjusted Spearman-type correlations will be used to evaluate cross-sectional associations by using probability-scale residuals, which yield a fully adjusted rank-based correlation metric [27]. These analyses will serve as sensitivity analyses complementing the longitudinal trajectory models.

### Secondary analyses

Secondary analyses are explicitly exploratory and include data-driven approaches, such as clustering and latent class modelling, to investigate heterogeneity in recovery trajectories and explore potential recovery phenotype groups, as well as temporal association analyses examining relationships between early and medium-term post-operative measures. All secondary analyses are exploratory and hypothesis-generating in nature, to inform future hypothesis-driven research, rather than to support confirmatory inference or predictive model development.

#### Secondary criteria 1: Exploring recovery-phenotype groups.

Daily step count profiles will be smoothed using locally estimated scatter-plot smoothing (LOESS) to reduce noise and emphasise underlying patterns, consistent with prior digital recovery analytics [28]. Shape-based k-means clustering will then be applied to these smoothed trajectories, exploring cluster solutions from 2–5 groups. Clustering results will be evaluated using silhouette indices and clinical interpretability.

Clustering methods will be used to identify descriptive recovery phenotypes. Latent trajectory models such as growth mixture models (GMMs) and group-based trajectory models (GBTMs) will be used to estimate latent classes representing underlying recovery patterns. These approaches are complementary, with clustering providing empirical grouping and latent models characterising probabilistic recovery structures.

Latent class model selection will follow established statistical guidelines, including AIC, BIC, entropy, and minimum class sizes [29]. GMMs will allow within-class variability. GBTMs will model homogeneous within-class trajectories.

#### Secondary criteria 2: Exploratory early-late temporal association analyses.

Early post-operative wearable-derived step count and PROMs will be examined to determine whether they are temporally associated with corresponding medium-term outcomes at 6 months. Mixed-effects regression models will be specified with fixed effects for time, the outcome measure, and a time × measure interaction to allow the magnitude of associations to vary across the post-operative follow-up period.

For within-variable temporal analyses (e.g., early step count in relation to subsequent step count), mixed-effects autoregressive model structures will be explored, such as stepₜ ~ stepₜ ₋ ₁ with participant-level random intercepts, to explicitly account for temporal dependence in repeated mobility measurements.

For between-variable temporal analyses, models will be adjusted for relevant baseline covariates which are specified in Table 4. Marginal effects will be estimated within the prespecified 7-day analytic windows to characterise how early post-operative outcome measures relate to medium-term outcomes measures.

**Table 4:** Overview of statistical analysis plan.

Research Question	Statistical methods	Outcomes	Covariates
Trend analysis of step count PROMs over time after surgery.	Smartwatch step counts: Linear Mixed Models (LMM) with log transformation, or binomial regression. OKS: random effect Tobit regression. EQ-5D-5L: random effect Tobit regression and beta binomial regression. Pain NRS: LMM or ordinal regression model All models will include random intercepts and time as a fixed effect, modelled using restricted cubic splines to capture non-linear trends over time.	Visualization of the predicted values (or predicted probabilities, where appropriate)	Baseline covariates (age, sex, BMI, baseline OKS, baseline EQ-5D-5L, baseline pain, hospital LOS, surgical method and participation in prehabilitation exercise classes.)
Cross-sectional associations between daily step count and OKS, EQ-5D-5L, pain numerical rating scale (NRS).	Covariate-adjusted Spearman approach based on probability-scale residuals at days 1–7, 60–67, 120–127 and 180–187 post-operative	Strength of associations between mobility and variables.	Baseline covariates (age, sex, BMI, baseline OKS, baseline EQ-5D-5L, baseline pain, hospital LOS and participation in prehabilitation exercise classes.)
To define group-based recovery trajectories based on step count after surgery	Unsupervised method: k-means clustering performed on the smoothed trajectories Latent class modelling approaches: Growth Mixture Modelling and Group-Based Trajectory Modelling.	Recovery phenotype groups based on step count trajectories	Latent class modelling approaches: Baseline covariates: age, sex, BMI, baseline OKS/pain.
Exploratory Early-Late Temporal Association Analyses	Smartwatch step counts: Linear Mixed Models (LMM) with log transformation, or binomial regression. OKS: random effect Tobit regression. EQ-5D-5L: random effect Tobit regression and beta binomial regression. Pain NRS: LMM or ordinal regression model All models will include random intercepts, time as a categorical fixed effect, and an interaction term with the principal independent factor (either step counts or PROMs) to assess how the effect of the predictor changes over time.	Regression coefficients and marginal effects will be reported at each time point to assess whether early post-operative measures contain sufficient temporal signal.	Baseline covariates: age, sex, BMI, baseline OKS/pain.

Analyses will be performed using R (v ≥ 4.3).

#### Compliance monitoring.

Compliance will be tracked across two key domains (1) wearable adherence (2) PROM completion.

#### Wearable adherence.

Participants will be instructed to wear the Garmin Vivosmart 5 continuously from up to four weeks pre-operatively to six months post-operatively, with daytime wear expected and night-time wear optional. Adherence will be monitored using the Labfront platform.

A valid day will require >12 hours of wear time between 07:00–22:00, based on evidence that shorter wear leads to biased mobility estimates. A valid week will require ≥3 such days, reflecting Mobilise-D findings that this threshold achieves reliable weekly mobility values without introducing excessive sample loss or selection bias [30]. To ensure adequate alignment between mobility measurement and clinical assessment, this study will incorporate a ± 14-day tolerance window around each PROM collection period. When wearable data do not fully cover the 7-day PROM window, mobility data may be drawn from up to 14 days before or after the scheduled assessment period to complete the sampling frame. This approach follows the methodology outlined in the Mobilise-D study protocol [11], which recommends allowing limited flexibility around clinical assessment dates to ensure sufficient real-world mobility data while maintaining appropriate temporal correspondence between digital and clinical outcomes.

Weeks with no valid days will be treated as missing. PROMs will be multiply imputed under a multilevel framework, while wearable summaries will not be imputed in the primary analysis; sensitivity analyses will include complete-case and model-based imputation approaches.

Implausible values will be excluded according to a priori rules: daily step counts >50,000; heart rate <30 bpm or >220 bpm sustained for >10 minutes; sleep duration <2 h or >14 h on valid-wear days; or HRV values outside the device reportable range or coinciding with non-wear segments. All exclusions will be logged. Sensitivity analyses will repeat primary models using winsorisation (1st-99th percentile) instead of exclusions.

Non-wear will be inferred from data availability within Labfront, defined by absence of step counts and heart-rate recordings over sustained periods, as the platform does not provide direct access to raw accelerometery or proprietary Garmin non-wear algorithms. Device firmware and Garmin app version updates will be documented manually, and analyses will include version-epoch indicators with stratified sensitivity analyses where algorithm changes occur. Periodic adherence feedback will be provided to participants.

#### PROM compliance.

Compliance will be summarised as the proportion of scheduled PROMs completed. Thresholds are set to guide participant support rather than to exclude data: a weekly target of ≥70% and a monthly target of ≥60% are considered realistic given the duration of follow-up. This aligns with evidence regarding participant adherence to PROMs, which shows completion rates fall between 50% to 80% [31]. All available PROM data will be included in the primary analyses; sensitivity analyses will compare results across adherence bands and under stricter completion thresholds.

Because missingness may be informative (e.g., participants with greater pain, fatigue, or complications may be less likely to complete PROMs), missing PROM data will be addressed using multilevel multiple imputation, which accounts for within-person correlation over time [32,33]. Sensitivity analyses will additionally explore departures from missing-at-random assumptions.

#### Analytical considerations.

Adherence metrics will be incorporated as covariates and explored in sensitivity analyses to assess their potential influence on recovery trajectories. Sub-group analyses will evaluate whether adherence varies systematically by demographic or clinical factors, recognising that missingness may be informative.

### Discussion

#### Bias and limitations.

As a single-centre investigation conducted at the Beacon Hospital, findings may have limited generalisability to other healthcare systems or rehabilitation settings. The observation window does not extend to long-term outcomes, where participant dissatisfaction often emerges more prominently [5–7]. Reliance on a consumer-grade device may introduce measurement error, although validation studies support acceptable accuracy for step count and heart rate under free-living conditions [34]. Firmware updates or proprietary algorithm changes may also affect data consistency; device versioning will be documented and sensitivity analyses undertaken where necessary. Finally, providing participants with adherence feedback may introduce reactivity bias, as behaviour can be influenced by awareness of monitoring.

#### Amendments and study termination.

Any substantive amendments to the study protocol, including changes to eligibility criteria, outcomes, statistical methods or data collection procedures, will be submitted for approval to the Beacon Hospital Research Ethics Committee prior to implementation. Amendments will also be documented and time-stamped on the OSF registration. Minor administrative changes that do not impact participant involvement or study methodology will be recorded in an internal amendment log. Early termination of the study will be considered only in the event of unforeseen safety concerns, logistical infeasibility, or withdrawal of institutional support. Should termination occur, all available data will be analysed to the extent possible, and a final report will be disseminated outlining the reasons for discontinuation and implications for interpretation.
